# Impact of *Weissella cibaria* BYL4.2 and its supernatants on *Penicillium chrysogenum* metabolism

**DOI:** 10.3389/fmicb.2022.983613

**Published:** 2022-10-05

**Authors:** Di Yao, Xiaoyu Wang, Lixue Ma, Mengna Wu, Lei Xu, Qiaoru Yu, Liyuan Zhang, Xiqun Zheng

**Affiliations:** Department of Food Science and Engineering, College of Food, Heilongjiang Bayi Agricultural University, Daqing, Heilongjiang, China

**Keywords:** antifungal effect, *Weissella cibaria*, *Penicillium chrysogenum*, LAB, metabolome

## Abstract

Lactic acid bacteria (LAB) can produce a vast spectrum of antifungal metabolites to inhibit fungal growth. The purpose of this study was to elucidate the antifungal effect of isolated *Weissella cibaria* BYL4.2 on *Penicillium chrysogenum*, the antifungal activity of *W. cibaria* BYL4.2 against *P. chrysogenum* was evaluated by the superposition method, results showed that it had obviously antifungal activity against *P. chrysogenum*. Studying the probiotic properties of BYL4.2 and determining it as beneficial bacteria. Furtherly, different treatments were carried out to characterize the antifungal activity of cell-free supernatant (CFS) produced by *W. cibaria* BYL4.2, and it was shown that the CFS was pH-dependent, partly heat-sensitive, and was not influenced by proteinaceous treatment. The CFS of *W. cibaria* BYL4.2 was analyzed by high-performance liquid chromatography (HPLC) and found the highest content of lactic acid. Screening of metabolic markers by a non-targeted metabolomics approach based liquid chromatography-mass spectrometry (LC-MS). The results speculated that organic acid especially detected D-tartaric acid was the main antifungal substance of CFS, which could cause the down-regulation of metabolites in the ABC transporters pathway, thereby inhibiting the growth of *P. chrysogenum*. Therefore, this study may provide important information for the inhibitory mechanism of *W. cibaria* BYL4.2 on *P. chrysogenum*, and provide a basis for further research on the antifungal effect of *Weissella.*

## Introduction

Filamentous fungi is a common contaminating fungus in food, which can cause spoilage and deterioration of dairy products, cereals, fruits and vegetables, and grains, reducing the quality of food and shortening the shelf life, thereby causing serious economic losses (Le Lay et al., 2016). Therefore, controlling filamentous fungi contamination is very pivotal. So far, different methods have been used to protect food and extend its shelf life, such as air treatment, modified atmosphere packaging and addition of chemical preservatives (propionate, sorbate, and ethanol). However, with the improvement of consumers’ quality of life, the importance of food safety has been paid more and more attention. Compared with chemical preservatives, natural preservatives have received extensive attention. As one of the natural preservatives, lactic acid bacteria (LAB) is a promising alternative to chemical preservatives (Chen H. et al., 2021). LAB are a class of non-pathogenic gram-positive bacteria that degrade fermentable carbohydrates and produce large amounts of lactic acid (Meng and Xu, 2019). Application studies have confirmed that LAB are beneficial microorganisms that can not only be used in industrial production but also are essential for human and animal bodies, which have physiological functions such as maintaining the ecological balance of intestinal flora, inhibiting pathogenic bacteria and fungal, promoting the decomposition of nutrients, etc (Wang et al., 2019). Many studies have shown that LAB have antibacterial and antifungal effects, so have been widely concerned as a safe biological preservative. *Lactobacillus casei* HQ201 was screened from fermented products, which has an inhibitory effect on some bacteria such as *Micrococcus luteus* and *Staphylococcus aureus* but has no inhibitory effect on mold and yeast (Yuan et al., 2019). The *Lactobacillus plantarum* IMAU10014 was isolated from milk wine and had good antifungal activity against *Penicillium citrinum*, *Penicillium digitatum*, *Fusarium oxysporum*, and other fungi (Wang et al., 2012). So, it is necessary to further explore the antifungal mechanism of LAB.

Studies indicated that LAB can produce a variety of antifungal metabolites during the growth process. Many of the antifungal substances of LAB have been proven to have antifungal properties, such as organic acids, ethanol, hydrogen peroxide, bacteriocins, etc., these antifungal and bacterial substances have a synergistic effect, making the cell-free supernatant (CFS) of LAB has a very good inhibitory effect on pathogenic bacteria and fungi (Kanižai Šaric et al., 2021; Wang et al., 2021). Currently, liquid chromatography-mass spectrometry (LC-MS) is a powerful and valuable emerging tool for analyzing the metabolites of microorganisms (Wang and Xie, 2020). Non-targeted metabolomics involves the simultaneous detection of multiple endogenous metabolites and provides an unbiased and global metabolite spectrum of microorganisms by LC-MS. Through quantitative analysis, small-molecule metabolites, including amino acids, peptides, complex carbohydrates, lipids, organic acids, and nucleotides can be separated (Want et al., 2010). The peptide produced by *L. plantarum* TE10 was detected that has antifungal activity against the spoilage fungus *Aspergillus flavus* MD3 using LC-MS/MS (Muhialdin et al., 2020). The CFS of LAB was analyzed by high-performance liquid chromatography (HPLC) and LC-MS, which found that the main antifungal compounds against *Penicillium expansum* were organic acids and carboxylic acids (Chen Q. et al., 2021). The antifungal metabolites produced by *Paenibacillus* sp. MS2379 was characterized by a comprehensive method of ultra-high-performance liquid chromatography coupled with quadrupole time-of-flight mass spectrometry (UHPLC-QTOF-MS) analysis and nuclear magnetic resonance spectroscopy interpretation (Qiu et al., 2018). However, many previous studies have focused on the antifungal substances of different LAB, to date, the detection of changes in the content of specific metabolites and biosynthetic pathways after co-culture of LAB with fungi has been limited. And compared with bacteria, there are relatively few studies on LAB that inhibit fungi.

Therefore, it is necessary to study the metabolic pathway of LAB against fungi and further explore the mechanism of the antifungal effect. The filamentous fungi used in this study is *Penicillium chrysogenum* isolated from corn preserved in our laboratory. No studies have been done on *Weissella cibaria* suppressing *P. chrysogenum*, so to identify and investigate the antifungal activity of *W. cibaria* BYL4.2 isolated from kimchi, we characterized antifungal compounds and analyzed antifungal metabolites in CFS qualitatively and quantitatively. The composition, pathways and changes of metabolites inhibiting *P. chrysogenum* by *W. cibaria* BYL4.2 were investigated by LC-MS non-targeted metabolomics method. Statistical analysis was performed to explore substantial differences between *P. chrysogenum* (P groups) and *P. chrysogenum* + *W. cibaria* BYL4.2 (L_P groups). Through analysis of the metabolic mechanism of *W. cibaria* BYL4.2 inhibition of *P. chrysogenum* provides a basis for the study of LAB inhibiting fungi.

## Materials and methods

### Isolation and identification of lactic acid bacteria with antifungal activity

The kimchi samples were diluted with physiological saline, cultured on de man rogosa and sharpe (MRS) agar (HB0384, Hopebiol, China) or broth (HB0384-1, Hopebiol, China) at 37°C for 48 h, and subcultured three times. The purified colonies were selected for hemolysis test, gram staining and microscopy, and the morphology of the colonies was observed under an optical microscope. Meanwhile, *P. chrysogenum* isolated from moldy corn seeds and stored in the laboratory was inoculated on potato dextrose agar (PDA) (HB0233, Hopebiol, China) and cultured at 28°C for 3 days. Sterile water was poured to prepare spore suspension, then the concentration of the spore suspension was adjusted to 10^7^ spores/ml.

The antifungal activity of the strain was determined by agar overlay method. Briefly, the LAB isolates were inoculated in 5-cm lines on MRS agar plates and incubated at 37°C for 24 h. The plates were then overlaid with 10 ml of PDA containing 10^7^ spores/ml of *P. chrysogenum*. Clear zones of inhibition were observed on the plates after 3 days of incubation at 28°C. The LAB isolate showing significant antifungal activity was selected for further studies.

The selected LAB isolate BYL4.2 was characterized genotypically based on 16S rRNA sequencing. The genomic DNA isolated using Ezup column bacteria genomic DNA purification kit (B518255, Sangon Biotech, China) was subjected to 16S rRNA amplification using the universal primers 27F and 1492R. The PCR product was purified using the SanPrep Column PCR Product Purification Kit (B518141, Sangon Biotech, China) and sequenced (Comate Bioscience Co., Ltd., Jilin, China). The generated sequence was analyzed using the National Center for Biotechnology Information (NCBI) BLAST tool. The sequences of *W. cibaria* BYL4.2 and other representative strains were aligned using the computer software MEGA X and the phylogenetic tree was constructed using the neighbor-joining method.

### Probiotic characterization of *Weissella cibaria* BYL4.2

The growth kinetics and acidification curve of *W. cibaria* BYL4.2 were measured. The strain was inoculated in MRS broth and cultured at 37°C for 24 h. The absorbance of 600 nm and pH were determined every 2 h. To determine antibiotic susceptibility, 100 μl of the BYL4.2 liquid culture was spread on MRS agar. The test papers of drug sensibility were attached to the surface of the medium, and the diameter of each inhibition zone was measured after culturing at 37°C for 24 h.

The inhibitory effect of *W. cibaria* BYL4.2 on pathogenic bacteria was determined by double-layer agar diffusion method. First, the Oxford cups were evenly placed on the solidified 0.5% water agar, and 20 ml LB agar containing indicator bacteria was poured into it. A 0.2 ml of BYL4.2 liquid culture obtained after centrifugation (8,000 rpm, 10 min, 4°C) were added to the formed wells, and the diameter of the inhibition zones was measured after culturing at 37°C for 24 h.

### Antifungal activity of *Weissella cibaria* BYL4.2-CFS

The liquid culture of *W. cibaria* BYL4.2 in the stable phase was centrifuged (8,000 rpm, 15 min, 4°C), and filtered with 0.22 μm membranes (SLGV004SL, Millipore, MA, United States) to obtain the CFS. Determination of the antifungal effect of CFS by Oxford cups agar diffusion method. The plates were overlaid with 10 ml of PDA containing 10^7^ spores/ml of *P. chrysogenum*, place the Oxford cup evenly on the medium after it solidifies, then add 200 μl of BYL4.2-CFS to the Oxford cup, respectively. The equivalent MRS medium was used instead of BYL4.2-CFS as a control, cultured at 28°C for 3 days, and the size of the inhibition zone was observed to determine the antifungal effect of BYL4.2-CFS on *P. chrysogenum*.

Previous research has shown the stability of the antifungal activity of *W. cibaria* BYL4.2 was characterized based on the sensitivity of the CFS toward changes in pH, heat, and lytic enzymes (Sangmanee and Hongpattarakere, 2014). CFS was subjected to different conditions of heat treatment, acid treatment and protease action, and the antifungal stability of *W. cibaria* BYL4.2-CFS was compared using a 96-well microtiter plate (Chen H. et al., 2021). The CFS was tested for its susceptibility to catalase, trypsin, pepsin and proteinase K (2 mg/ml) treatment incubated at 37°C for 2 h (Yuanye, Shanghai, China), followed by enzyme inactivation at 100°C for 5 min. To assess the thermal stability, CFS was heated at 60, 80, 100, and 121°C for 20 min, then cooled to room temperature. The effect of pH on CFS was tested by adjusting the pH to 3.0, 4.0, 5.0, 6.0, and 7.0 using sterile 1 M NaOH or 1 M HCl, the residual antifungal activity was determined by 96-well microtiter plates (701011, NEST, China) and the untreated CFS was used as the control. Briefly, 140 μl of the treatmented CFS was added to 10 μl of the spore suspension (10^7^ spores/ml) in each well. The positive control (PC) was 10 μl of the spore suspension (10^7^ spores/ml) inoculated in 140 μl of fresh MRS broth in each well. Then, 150 μl of sterile fresh MRS broth was added to each well. The microplates were then incubated at 28°C for 72 h. All samples were analyzed in triplicate. The inhibition rates were calculated as a percentage calculation of the PC values subtracted out the OD_600__*nm*_ values obtained from *P. chrysogenum* growth in the CFS and then divide by the PC.

### Determination of *Weissella cibaria* BYL4.2-CFS organic acid by high-performance liquid chromatography

Aspirated 5 ml of *W. cibaria* BYL4.2-CFS and 1 ml of 25% (v/v) metaphosphoric acid (37267-86-0, Sinopharm Chemical Reagent Co., Ltd., China) solution to fully mix and frozen at −20°C overnight. After thawing, the samples were centrifuged (12,000 rpm, 10 min); the supernatant was filtered by 0.22 μm filter membranes, and the filtrate was analyzed by HPLC (Agilent 1,200, Agilent Technologies Co., Ltd., CA, USA). Qualitative by retention time of standard substance, quantitative by external standard method of peak area. Chromatographic column: Sepax Carbomix H-NP: 7.8 mm×300 mm, 5 μm particle size; column temperature: 60°C; mobile phase: 2.5 mM aqueous sulfuric acid, containing 5% acetonitrile (v/v); flow rate: 0.50 ml/min; Detector: Differential refractive index detector.

### Metabolome analysis by liquid chromatography-mass spectrometry

1*Penicillium chrysogenum* was inoculated with 1% inoculum (10^7^ spores/ml) into a conical flask containing 50 ml of potato dextrose broth (PDB) as control groups (P); *P. chrysogenum* was inoculated into a conical flask containing 40 ml of PDB with 1% inoculation amount (10^7^ spores/ml) then added in 10 ml of *W. cibaria* BYL4.2-CFS as co-culture groups (L_P), which were incubated in a constant temperature shaker (Ruihua, Changzhou, China) for 3 days (120 rpm, 28°C). Considering that PDB as a fungi medium may have an impact on the fermentation of *W. cibaria* BYL4.2-CFS, so *W. cibaria* BYL4.2-CFS was added to PDB (PDB_L), PDB as the control groups. The hyphae of L_P and P groups were collected after filtration with nine layers of sterile gauze.

A total of 200 μl PDB_L and PDB were transferred into 1.5 ml centrifuge tubes; 800 μl of extract [methanol: acetonitrile = 1:1 (v: v)] which containing 0.02 mg/ml internal standard (L-2-chlorophenylalanine) was added; after vortex mixing for 30 s, extracted by cryogenic ultrasound for 30 min (5°C, 40 kHz); the samples were placed at −20°C for 30 min; after centrifugation for 15 min (13,000 g, 4°C), the supernatant was dried with nitrogen; 120 μl of reconstituted solution (acetonitrile: water = 1:1) was added to reconstitute; vortexed for 30 s, extracted by low-temperature ultrasound for 5 min (5°C, 40 kHz).

A total of 50 mg of the thallus of L_P and P were weighed into 2 ml centrifuge tubes, and a 6 mm diameter grinding ball and 400 μl extract [methanol: water = 4:1 (v: v)] containing 0.02 mg/ml internal standard were added; the frozen tissue grinder was ground for 6 min (−10°C, 50 Hz); extracted with low-temperature ultrasound for 30 min (5°C, 40 kHz).

The samples were placed (−20°C, 30 min) and centrifuged for 15 min (13,000 g, 4°C), the supernatant was pipetted into injection vials for on-board analysis. In addition, 20 μl of the supernatant was pipetted from each sample and mixed as a quality control sample. The instrument platform for this LC-MS analysis was the ultra-high-performance liquid chromatography coupled with hybrid triple quadrupole (UHPLC-Q) Exactive HF-X system (Thermo Fisher Scientific, Massachusetts, USA). Chromatographic conditions: column ACQUITY UPLC HSS T3 (100 mm × 2.1 mm i.d., 1.8 μm; Waters, Milford, Massachusetts, USA); mobile phase A was 95% water (7732-18-5, Fisher Chemical, MA, USA) and 5% acetonitrile (75-05-8, Fisher Chemical, MA, USA) (containing 0.1% formic acid), mobile phase B was 47.5% acetonitrile and 47.5% isopropanol (67-63-0, CNW, Germany)and 5% water [containing 0.1% formic acid (64-18-6, CNW, Germany)], the injection volume was 2 μl, and the column temperature was 40°C. The samples were ionized by electrospray, and mass spectral signals were collected in positive and negative ion scanning modes, respectively.

### Statistical analysis

The data were analyzed statistically by the SPSS 22.0 and charted figures by OriginPro 2021. Analysis of variance (ANOVA, Duncan’s method at a significance level of *p* < 0.05) was applied to analyze the antifungal activities of CFS. Multivariate data analysis included principal components analysis (PCA) and partial least squares discriminant analysis (PLS-DA). The student’s test combined with the multivariate analysis of PLS-DA was used to evaluate the differential metabolites or potential marker (VIP > 1, *p* < 0.05). For analysis software, the online platform of Majorbio ISanger Cloud platform^1^ was used.

## Results and discussion

### Isolation, screening, and molecular identification of lactic acid bacteria isolates

**FIGURE 1:**
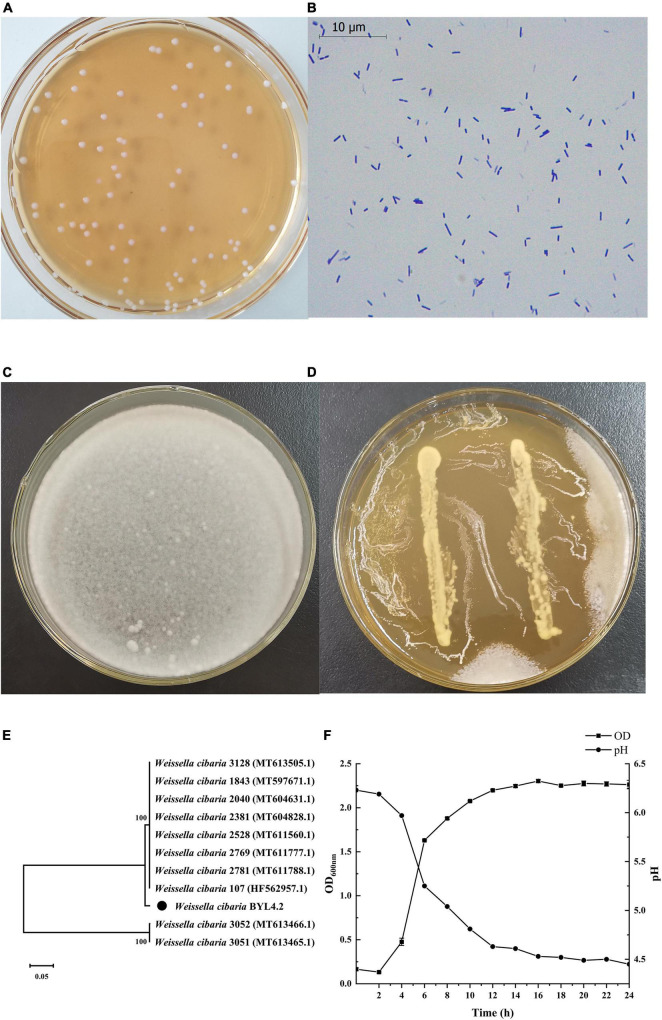
Identification and screening of antifungal LAB. The colony picture **(A)** and microscopic picture (×1,000) **(B)** of LAB isolates. Inhibition of *Penicillium chrysogenum* by *Weissella cibaria* BYL4.2 by agar overlay method after 3 day incubation at 28°C. Control *P. chrysogenum* **(C)**. *W. cibaria* BYL4.2 **(D)** overlaid by *P. chrysogenum*. Phylogenetic tree analysis of *W. cibaria* BYL4.2 using the neighbor-joining algorithm. The numbers above the branches are confidence limits expressed as percentages. The scale bar represents 0.05% sequence divergence **(E)**. Growth kinetics (square) and acidification profile (circle) of *W. cibaria* BYL4.2 in MRS broth at 37°C for 24 h **(F)**.

About 30 strains were isolated from the kimchi samples. Compared with the control groups and other isolates strain, BYL4.2 with rod-shaped and gram-positive (Table 1 and Figures 1A,B) had a significant inhibitory effect on *P. chrysogenum* (Figures 1C,D). The phylogenetic tree analysis was carried out to verify the isolate’s evolutionary position, which was constructed based on 16S rRNA sequences of GenBank database (Figure 1E). The BYL4.2 isolate was identified as *W. cibaria* through sequencing of its 16S rRNA amplicon and sequence alignment (100% identity). Early studies in this field have preliminarily shown the *Weissella* strains have antifungal activity, have varying degrees of inhibitory effect on *Verticillium wilt*, *Mucor folium*, etc (Quattrini et al., 2020). It is shown that *Weissella* can inhibit the growth of filamentous fungi, which has a potentially important role for *Weissella* as a bacteriostatic agent.

**TABLE 1:** The probiotic properties of *Weissella cibaria* BYL4.2.

Gram staining	Gram-positive rods	
Hemolytic test	Negative	

**Antimicrobial activity**	**Strain**	**Zone of inhibition (mm)**
	*Escherichia coli*	25.33 ± 0.58^a^
	*Staphylococcus aureus*	22.67 ± 1.15^b^
	*Pseudomonas aeruginosa*	18.00 ± 0.00^c^
	*Listeria monocytogenes*	17.67 ± 0.58^c^

**Antibiotics resistance**	**Antibiotics**	Diameter of bacteriostatic circle of *W. cibaria* BYL4.2* (mm)

	Penicillin	7.00 ± 0.00 (R)
	Streptomycin	9.00 ± 1.00 (R)
	Gentamicin	9.33 ± 1.15 (R)
	Erythromycin	22.33 ± 2.31 (S)
	Rifampicin	18.67 ± 1.15 (S)
	Cefotaxime	7.00 ± 0.00 (R)

*(S) and (R): susceptible and resistant, as per EFSA guidelines. Data shown are mean ± SD of triplicate values of independent experiments. Means with different superscript letters are significantly different horizontally (*P* < 0.05).

### Probiotic properties of BYL4.2

The acid-producing performance and growth rate of LAB are one of the criteria to measure whether they have the potential coming excellent strains. The growth rate of *W. cibaria* BYL4.2 is fast, and they quickly become the dominant strains in the fermentation environment, thereby preventing the reproduction of pathogenic bacteria; strong acid-producing ability, can quickly produce a large amount of organic acids such as lactic acid to form a low pH environment and inhibit the growth of harmful bacteria (He, 2021). As shown in Figure 1F, it can be seen that the lag period of *W. cibaria* BYL4.2 is short, entering the logarithmic growth phase at 2 h, and entering the stable phase at about 12 h. The research results of Cheng et al. (2022) showed that LAB generally enter the logarithmic growth stage 12–24 h after inoculation (Cheng et al., 2022). The results of this study are different from our experiment, which may be due to differences in strains. According to the pH change curve, with the growth, the pH decreased from 6.23 to 4.45 during 24 h. Previous studies had shown that LAB had excellent acid resistance and acid production capacity (Luo et al., 2021). Therefore, the strain in this study has a rapid growth rate and excellent acid production capacity and has the potential to be used as excellent fermentation inoculants.

In previous studies, the antifungal substances of LAB can secrete and produce are mainly organic acids, fatty acids, bacteriocins, hydrogen peroxide, carbon dioxide, cyclic dipeptide, and other antibacterial substances during the growth process, which can inhibit the growth of pathogenic *Escherichia coli*, *Staphylococcus aureus*, *Streptococcus suis*, etc., this feature promotes the intestinal health of humans and animals (Jang et al., 2016; Wang et al., 2020; Li and Gong, 2022). As shown in Table 1, *W. cibaria* BYL4.2 has inhibitory effects on *E. coli*, *S. aureus*, *Pseudomonas aeruginosa*, and *Listeria monocytogenes*, and the inhibition zones are 25.33 ± 0.58, 22.67 ± 1.15, 18.00 ± 0.00, and 17.67 ± 0.58, respectively. The inhibitory effect on *E. coli* is the strongest, followed by *S. aureus*, and the effect on *P. aeruginosa* and *L. monocytogenes* is similar. Compared with the other related research results of LAB inhibiting *E. coli* (9–16 mm), *S. aureus* (10–15 mm), *P. aeruginosa* (11–20 mm), and *L. monocytogenes* (13–18 mm), BYL4.2 has a better bacteriostatic effect on the four pathogenic bacteria (Zhu et al., 2019; Ye et al., 2021; Shi et al., 2022). Therefore, it is inferred that *W. cibaria* BYL4.2 has a significant inhibitory effect on these pathogens although the degree of inhibition varies.

Over the years the focus has been on the beneficial aspects of the probiotics due to their abundance in fermented foods, with antibiotic resistance being one of their important probiotic properties (Ojha et al., 2021). Recent studies have shown that different *W. cibaria* have different susceptibility to antibiotics (Wang et al., 2018; Kang et al., 2019). The *W. cibaria* JW15 was resistant to kanamycin and vancomycin and was sensitive to other antibiotics (Jang et al., 2021). In addition, hemolysin is a very common virulence factor among pathogens that frequently cause anemia and edema in the host (Nandi et al., 2016). The absence of hemolytic activity and antibiotic susceptibility are safety prerequisites for the selection of probiotic strains (Argyri et al., 2013). *W. cibaria* BYL4.2 was sensitive to erythromycin and rifampicin but resistant to penicillin, streptomycin, gentamicin, and cefotaxime. In addition, the tested strains were non-hemolytic (Table 1). Accordingly, we believe that *W. cibaria* BYL4.2 may be used as a potential commercial probiotic strain in the future.

### Antifungal activity of BYL4.2-CFS

**FIGURE 2:**
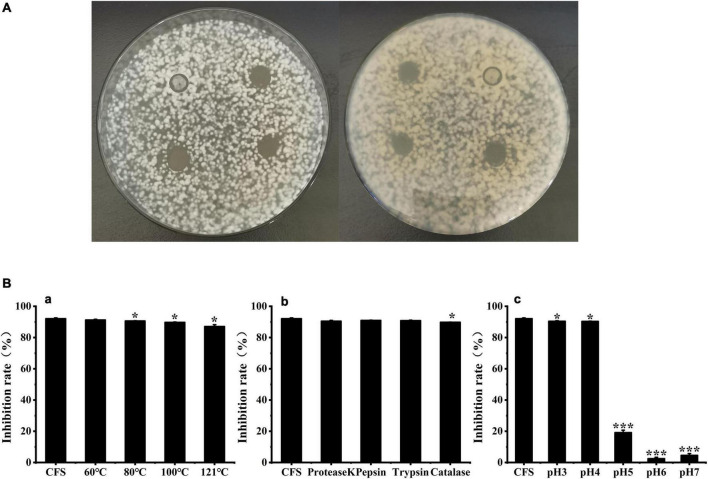
Effects of *Weissella cibaria* BYL4.2-CFS on the antifungal activity of *Penicillium chrysogenum*. Antifungal activity of BYL4.2-CFS in agar diffusion assay (the front and back) **(A)**. Antifungal effect after heat treatment **(a)**, acid treatment **(b)** and protease action **(c)**, and untreated *W. cibaria* BYL4.2-CFS as a control **(B)**. Data are means ± SDs; *p ≤ 0.05 and ***p ≤ 0.001.

Figure 2A shows that *W. cibaria* BYL4.2-CFS has a significant inhibitory effect on *P. chrysogenum* compared to the control. The CFS from BYL4.2-CFS reduced *P. chrysogenum* growth by inhibiting conidia germination, indicating that the antifungal activity is related to the bioactive compounds present in the CFS (Somashekaraiah et al., 2021). In order to study the composition of its antifungal substances, we preliminarily screened the main antifungal substances of BYL4.2-CFS by different treatment methods. As shown in Figure 2Ba, compared with untreated CFS, the antifungal effect after 60, 80, and 100°C treatment had no significant change; but the antifungal effect decreased significantly after treatment at 121°C. These results indicated that BYL4.2-CFS has thermal stability. Figure 2Bb showed that BYL4.2-CFS was sensitive to catalase, indicating that BYL4.2-CFS contains a small amount of hydrogen peroxide, which had an antifungal effect, but it was not the main antifungal substance. Figure 2Bc shows that compared with CFS, the antifungal effect at pH 5, 6, and 7 was significantly different, and decreased significantly when the pH increased. Researchers studied the factors (pH, temperature, and protease) affecting the antifungal activity of *Lactobacillus plantarum* XCT1-1 CFS, and found that the antifungal substances were more active under low pH conditions; this strain had good thermal stability, however, protease treatment had a great influence on the antifungal activity of the strain, so speculated that the antifungal active substances in XCT1-1-CFS were bacteriocins (Sun, 2020). According to reports, after treatment of *Levilactobacillus brevis* MYSN105-CFS with proteinase K, pH neutralization, and heat treatment, the antibacterial activity was moderately sensitive to proteinase K, with reduced activity at pH 7, while partial inactivation of CFS at 80°C within 20 min of heat treatment. The significant activity of *L. brevis* MYSN105 against *Fusarium verticillioides* could be attributed to the combined effects of a variety of organic acids (Somashekaraiah et al., 2021). To sum up, it is speculated that the main antifungal substances in *W. cibaria* BYL4.2-CFS are organic acid metabolites produced by it.

### Detection of organic acids of BYL4.2-CFS

In this study, 10 organic acids were quantitatively analyzed in *W. cibaria* BYL4.2-CFS by HPLC (Table 2). The results showed that the contents of citric acid, tartaric acid, lactic acid, acetic acid, and propionic acid were relatively high in *W. cibaria* BYL4.2-CFS, among which the content of lactic acid was the highest (9.55 ± 0.04), and the content of butyric acid was the lowest, almost none. Analogous, others also detected high levels of citric acid, lactic acid, and acetic acid in LAB (Jiang et al., 2020; Li et al., 2020). Contacting above, this suggests these organic acids may inhibit fungal growth through synergistic or additive effects.

**TABLE 2:** The organic acids produced in the *Weissella cibaria* BYL4.2-CFS was quantitatively determined by HPLC.

Compounds	*W. cibaria* BYL4.2-CFS (g/L)	Compounds	*W. cibaria* BYL4.2-CFS (g/L)
Citric acid	5.21 ± 0.03^b^	Formic acid	0.52 ± 0.02^f^
D-tartaric acid	0.61 ± 0.01^e^	Acetic acid	3.73 ± 0.02^c^
D-malic acid	0.08 ± 0.00^h^	Propionic acid	0.80 ± 0.01^d^
Succinic acid	0.05 ± 0.00^h^	Isobutyric acid	0.12 ± 0.00^g^
Lactic acid	9.55 ± 0.04^a^	Butyric acid	0.02 ± 0.01^i^

Data shown are mean ± SD obtained across triplicate measurements. Means with different superscript letters are significantly different horizontally (P < 0.05).

### Metabolites compounds by liquid chromatography-mass spectrometry

**FIGURE 3:**
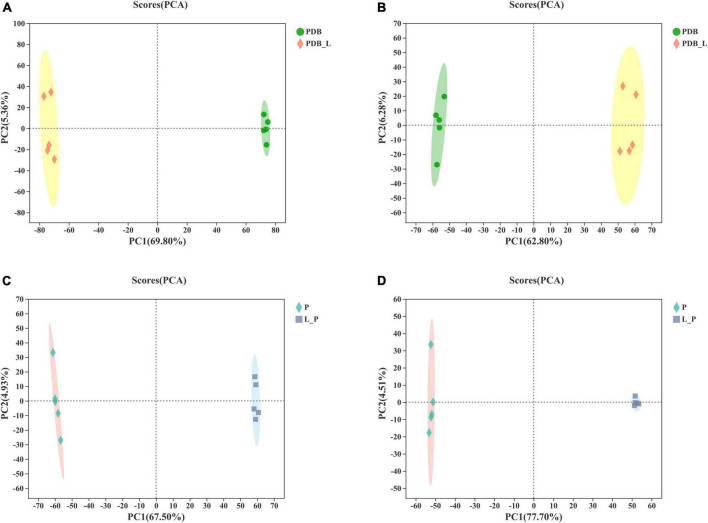
The score scatter plot of principal component analysis (PCA). PDB and *Weissella cibaria* BYL4.2-CFS + PDB of positive ion mode **(A)** and negative ion mode **(B)** and *Penicillium chrysogenum* and *W. cibaria* BYL4.2-CFS + *P. chrysogenum* of positive ion mode **(C)** and negative ion mode **(D)**. PDB, PDB; PDB_L, *W. cibaria* BYL4.2-CFS + PDB; P, *P. chrysogenum*; L_P, *W. cibaria* BYL4.2-CFS + *P. chrysogenum*.

Based on LC-MS detection of LAB-CFS and intracellular metabolites of fungal, the PCA method was used to analyze the PDB_L and PDB groups, the L_P and P groups to grasp the overall situation of the metabolites (Figure 3 and Supplementary Table 1). The PCA score plot based on the first two principal components explains the difference in distribution between these groups, positive mode (PC1 69.80% and PC2 5.36%) and negative mode (PC1 62.80% and PC2 6.28%) of PDB_L and PDB groups (Figures 3A,B), positive mode (PC1 67.50% and PC2 4.93%) and negative mode (PC1 77.70% and PC2 4.51%) of L_P and P groups (Figures 3C,D), respectively. These results showed that the PCA model was stable and reliable, the confidence intervals between the samples were all within 95%, with good biological repeatability of the samples. The principal components of the samples in these groups were significantly different, which were located in the positive and negative quadrants respectively, and can be used for subsequent analysis. A previous study showed that the metabolites observed in bacterial and mold co-cultures were significantly different from those observed in their respective monocultures (Swift et al., 2021). It could be seen from Figure 3 that there was a significant difference between the PDB_L and PDB groups, indicating that after *W. cibaria* BYL4.2-CFS was added to PDB, the composition and content of metabolites changed significantly. Furthermore, there was also a significant difference between the L_P and P groups, indicating that *P. chrysogenum* was inhibited and the content and composition of metabolites would be changed by BYL4.2-CFS.

**FIGURE 4:**
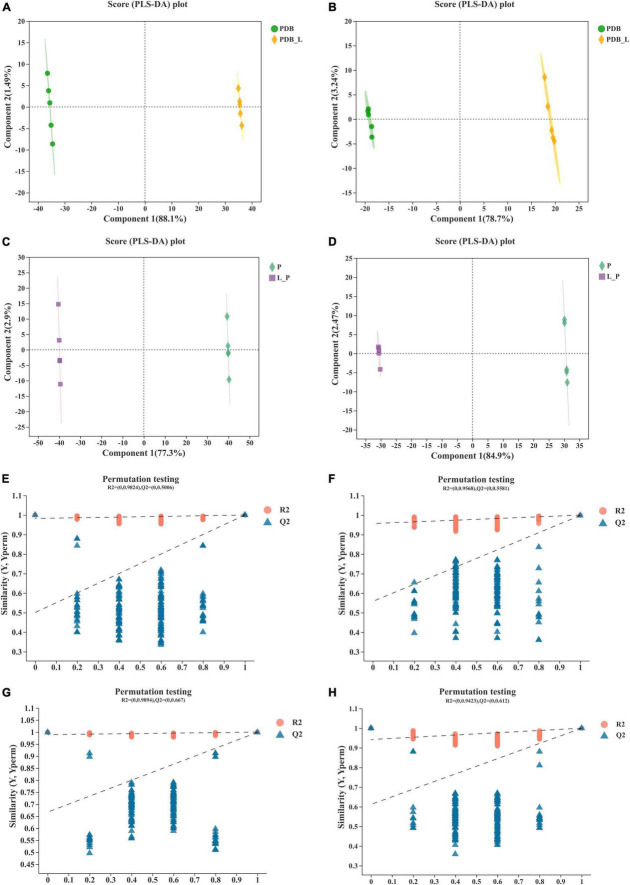
Validation of partial least squares discriminant analysis (PLS-DA) models. Pairwise comparation of among PDB, PDB_L, L_P, and P in positive ion mode **(A,C,E,G)** and negative ion mode **(B,D,F,H)**.

To further identify the relevant metabolites responsible for groups segregation, PLS-DA-based pairwise comparison method should be used to show co-culture metabolomics differences (Chen et al., 2020). The PLS-DA model showed strong goodness of fit (R^2^X) and high predictability (Q^2^). All PLS-DA models were validated by the Response Permutation Test (RPT), which showed no overfitting and no false positives in the data (Supplementary Table 1). In addition, permutation test cross-validation was performed 200 times to ensure model suitability. With the decrease of permutation retention, R^2^ and Q^2^ decreased, and the regression line showed an upward trend, indicating that the permutation test passed and the model no had overfitting (Figure 4). The clear clusters revealed metabolite differences between the PDB_L and PDB, L_P, and P groups.

**FIGURE 5:**
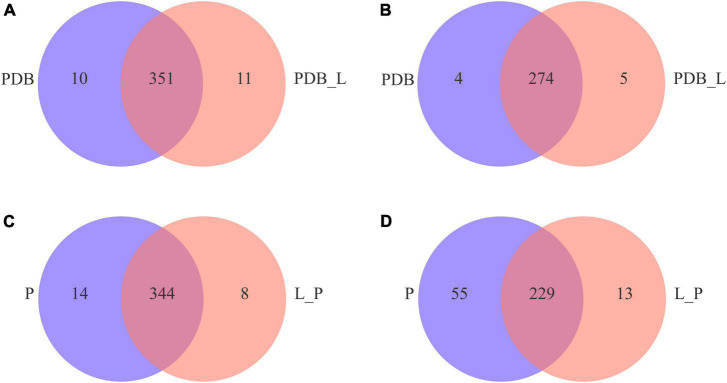
Venn diagrams of positive **(A,C)** and negative **(B,D)** ion modes of differential metabolites in PDB_L vs. PDB and L_P vs. P groups.

For better interpretation and visualization of the data (Tor-Roca et al., 2020), Figure 5 is a Venn diagram of metabolites between PDB_L vs. PDB and L_P vs. P groups. The results showed that there are 351 shared metabolites by PDB_L and PDB in positive ion mode (Figure 5A), and 344 for L_P and P (Figure 5C); while in negative ion mode, there are 274 shared metabolites by PDB_L and PDB (Figure 5B), and 229 for L_P and P (Figure 5D). It can be seen that there are more shared metabolites in the positive ion mode than in the negative ion mode. From the figure, it could be inferred that the unique metabolites in PDB_L and L_P are partial metabolites of *W. cibaria* BYL4.2-CFS, with a total of 37 metabolites, including 6 overlapping metabolites (Supplementary Table 2). Among them, the acids were 6-[(4,5-dihydroxy-7-methoxy-2,2-dimethyl-3,4-dihydro-2h-1-be nzopyran-6-yl)oxy]-3,4,5-trihydroxyoxane-2-carboxylic acid, 3,4-methylene suberic acid, 12-hydroxyheptadecanoic acid and tall oil fatty acid (TOFA). TOFA inhibited the fatty acid metabolism, and it could also inhibit human tumor cells (Qiu et al., 2019). These acids may be considered organic acids contained in *W. cibaria* BYL4.2, thus speculating that they may have an important role in inhibiting the growth of fungi.

### Quantitative and class identification results of differential metabolites

**FIGURE 6:**
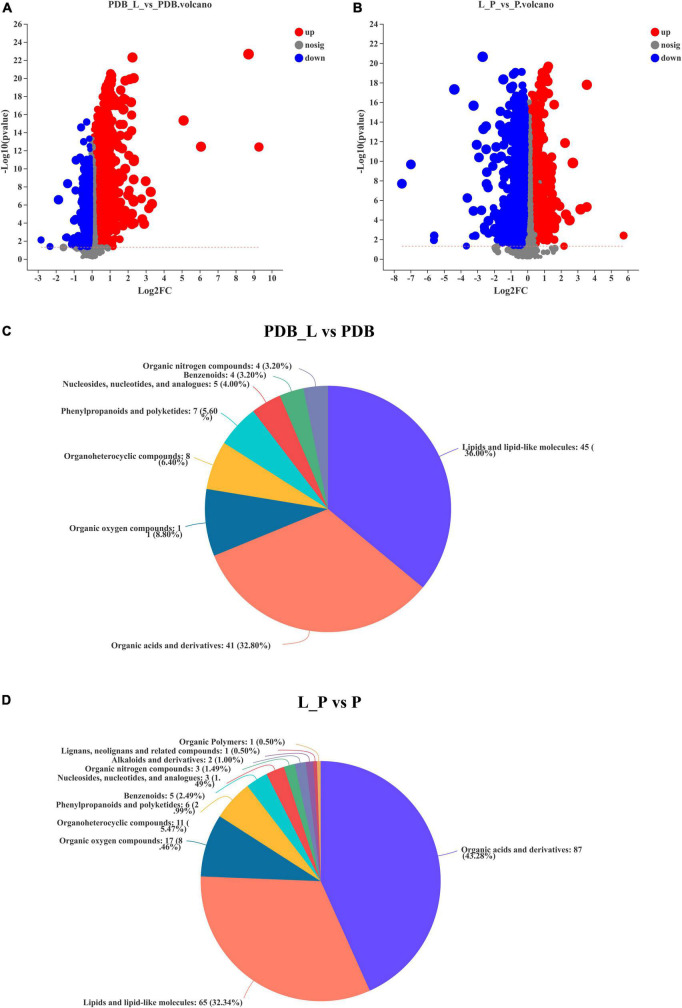
Volcano plot of differential metabolites and classification of the Human Metabolome Database (HMDB) compounds. **(A,C)** PDB_L vs. PDB groups; **(B,D)** L_P vs. P groups, red is up-regulation, blue is down-regulation (FC > 1.2 or <1.2, *p* < 0.05).

Figures 6A,B is a volcano plot of all differential metabolites in the PDB_L vs. PDB and L_P vs. P groups. Among them, *T*-test (*p* < 0.05) was used to screen differential metabolites according to fold change value (FC > 1.2 and FC < 1.2). It can be seen from the figure that compared with PDB, PDB_L had more up-regulated metabolites, and L_P had more down-regulated metabolites than P. Supplementary Tables 3, 4 summarized the being named differential metabolites in the PDB_L vs. PDB and L_P vs. P groups. In PDB_L vs. PDB, 145 named differential metabolites were analyzed including 68 cations and 77 anions. Compared to PDB groups, 76 differential metabolites were up-regulated and 69 were down-regulated in PDB_L groups (Supplementary Table 3). In L_P vs. P, 227 differential metabolites were analyzed, 99 cations and 128 anions. Compared to P groups, 43 metabolites were up-regulated and 184 were down-regulated in L_P groups (Supplementary Table 4).

The relative abundance of metabolites in the PDB_L vs. PDB and L_P vs. P groups is shown in Figures 6C,D, reflecting the content and intensity distribution of the differential metabolites. The differential metabolites in PDB_L vs. PDB groups mainly include 45 lipids and lipid-like molecules (36.00%, bile acids, alcohols and derivatives; steroid lactones; fatty acids and conjugates, etc.); 41 organic acids and derivatives (32.80%, amino acids, peptides, and analogs; beta hydroxy acids and derivatives; carboximidic acids; hybrid peptides; short-chain keto acids and derivatives; tricarboxylic acids and derivatives); 11 organic oxygen compounds (8.80%, alcohols and polyols; carbohydrates and carbohydrate conjugates); 8 organoheterocyclic compounds (6.40%, furoic acid and derivatives; gamma butyrolactones; imidazolidines; indolyl carboxylic acids and derivatives; purines and purine derivatives); 7 phenylpropanoids and polyketides (5.60%, cyclic diarylheptanoids); 5 nucleosides, nucleotides, and analogs (4.00%, pyrimidine ribonucleotides); 4 benzenoids (3.20%, anisoles; benzoyl derivatives); 4 organic nitrogen compounds (3.20%, amines; guanidines; quaternary ammonium salts) (Figure 6C and Supplementary Table 3).

The differential metabolites in L_P vs. P groups mainly include 87 organic acids and derivatives (43.28%, beta hydroxy acids and derivatives; amino acids, peptides, and analogs; sulfuric acid esters; organosulfonic acids and derivatives; hybrid peptides); 65 lipids and lipid-like molecules (32.34%, bile acids, alcohols and derivatives; fatty acids and conjugates; steroid lactones; terpenes);17 organic oxygen compounds (8.46%, alcohols and polyols; carbohydrates and carbohydrate conjugates; carbonyl compounds); 11 organoheterocyclic compounds (5.47%, pyrroloindoles; bilirubins; pyrimidines and pyrimidine derivatives; purines and purine derivatives, etc.); 6 phenylpropanoids and polyketides (2.99%, stilbene glycosides; hydroxycinnamic acids and derivatives); 5 benzenoids (2.49%, benzenesulfonic acids and derivatives; benzenesulfonamides; methoxybenzenes); 3 nucleosides, nucleotides, and analogs [1.49% (3′− > 5′)-dinucleotides; 5′-deoxy-5′-thionucleosides]; 3 organic nitrogen compounds (1.49%, amines; guanidines; quaternary ammonium salts); 2 alkaloids and derivatives (1.00%, chaetoglobosins); 1 lignans, neolignans and related compounds (0.50%); 1 organic polymers (0.50%) (Figure 6D and Supplementary Table 4). The latter three metabolites were present in the L_P vs. P groups but not in the PDB_L vs. PDB groups, but the abundance of these three substances is not high. The abundances of lipids and lipidoid molecules, as well as organic acids and their derivatives in PDB_L vs. PDB groups and L_P vs. P groups were higher, the difference was that lipids and lipidoid molecules were higher in PDB_L vs. PDB groups and the opposite in L_P vs. P groups. Increasing evidence and data suggest that lipid-like molecules, organic acids, and organoheterocyclic compounds were considered to be metabolites that had a significant impact on microbial growth (Chen et al., 2020; Seo et al., 2020).

**FIGURE 7:**
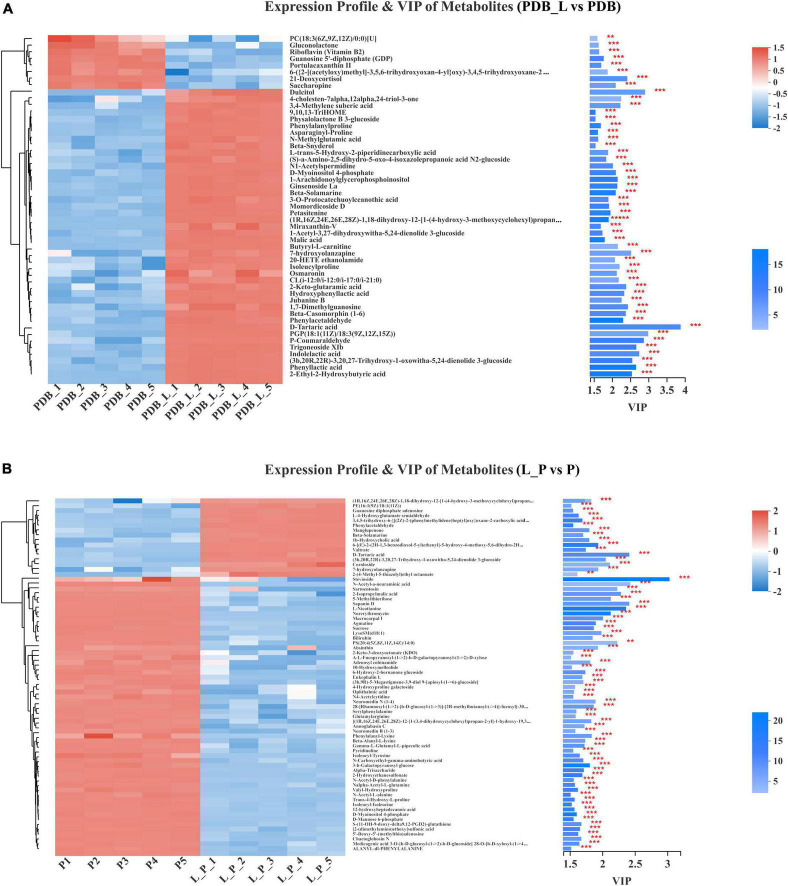
Comparison of the relative abundance of metabolites in PDB_L vs. PDB groups **(A)** and in L_P vs. P groups **(B)**. Levels of significance are defined as **p < 0.01 and ***p < 0.001.

The significant difference between the PDB_L vs. PDB groups and L_P vs. P groups metabolites was determined by the variable importance in the projection (VIP > 1.5), and *p*-value (*p* < 0.05). Figure 7 presented the differences in the relative levels of different metabolites identified by PDB_L vs. PDB groups and L_P vs. P groups. Heatmaps of the chemical compositions of PDB_L vs. PDB and L_P vs. P were drawn to show changes in metabolite concentrations (Figure 7). The PDB groups exhibited high content of lipids and lipid molecules, organic acids and derivatives, including 21-deoxycortisol and saccharopine (VIP > 2, *p* < 0.05) accounted for the majority. The abundance of lipids and lipid molecules were significantly up-regulated in the PDB_L groups compared with the PDB groups (VIP > 2.5, *p* < 0.01), including 2-ethyl-2-hydroxybutyric acid, trigoneoside XIb, (3b,20r,22r)-3,20,27-trihydroxy-1-oxowitha-5,24-dienolide 3-glucoside, PGP[18:1(11Z)/18:3(9Z,12Z,15Z)]. This indicated that some lipid content changed dramatically in the co-culture of PDB and *W. cibaria* BYL4.2-CFS, which directly improved the interactions by lipid metabolite (Figure 7A). However, the contents of 2-ethyl-2-hydroxybutyric acid, 2-keto-glutaramic acid, 3,4-methylene suberic acid, D-tartaric acid, phenyllactic acid, hydroxyphenyllactic acid, isoleucylproline, indolelactic acid, and other acidic substances in PDB_L were significantly up-regulated (Figure 7A), showing that *W. cibaria* BYL4.2-CFS was rich in organic acids.

As shown in Figure 7B, the P groups also exhibited high content of lipids and lipid-like molecules, including macrocarpal I, PS[20:4(5Z,8Z,11Z,14Z)/14:0], sarmentosin, 2-isopropylmalic acid, saponin D, stevioside; organic acids and derivatives including L-nicotianine; organic oxygen compounds including norerythromycin, N-acetyl-a-neuraminic acid, 5-methylthioribose (VIP > 2, *p* < 0.01). Macrocarpal I compounds have no bacteriostatic activity against gram-negative bacteria and fungi, but have bacteriostatic activity against gram-positive bacteria (Wei, 2015); PS[20:4(5Z,8Z,11Z,14Z)/14:0] is a glycerophospholipid that promotes the activation of glycerophospholipid metabolism and GPC pathway cycling (Wu et al., 2021). Sarmentosin is an unsaturated γ-hydroxynitrile glucoside produced by plants and is often chelated by lepidopterans, sarmentosin was previously reported only in plant systems and reported this metabolite for the first time in microbial systems (Jibrin et al., 2021). 2-isopropyl malic acid is an intermediate product of leucine biosynthesis (Suzuki et al., 2007). Saponin is a class of compounds that are ubiquitous in plants and composed of sapogenins and sugar groups, uronic acids or other organic acids (Xu et al., 2022). Stevia is a naturally occurring sweetener is Stevia, which is an excellent natural alternative to traditional sweeteners (Renjiao et al., 2021). The L_P groups exhibited high content of organic oxygen compounds, lipids and lipid molecules, including coroloside, D-tartaric acid and (3b,20R,22R)-3,20,27-trihydroxy-1-oxowitha-5,24-dienolide 3-glucoside (VIP > 2, *p* < 0.05). Similar to previous studies, metabolites were detected such as lipids and lipid-like molecules, organic oxygen compounds, organoheterocyclic compounds, nucleosides, nucleotides, and analogs in *P. chrysogenum* (Karpe et al., 2015). After co-culture, in L_P groups, D-tartaric acid, 1b-hydroxycholic acid and other acidic substances were up-regulated, yet 2-isopropylmalic acid, ophthalmic acid, n-carboxyethyl-gamma-aminobutyric acid, gamma-l-glutamyl-l-pipecolic acid, [2-(dimethylamino)ethoxy] sulfonic acid, 12-hydroxyheptadecanoic acid, etc., were down-regulated. The contribution of D-tartaric acid was the largest obviously in both PDB_L and L_P. Meanwhile, malic acid was also detected, but its contribution was smaller than that of D-tartaric acid in PDB_L. D-tartaric acid also was detected from *P. expansum* WH-3 (Bao et al., 2020). These results speculated that D-tartaric acid was the main organic acid that inhibits fungi.

### Functional annotation and enrichment analysis of metabolic pathways for differential metabolites

**FIGURE 8:**
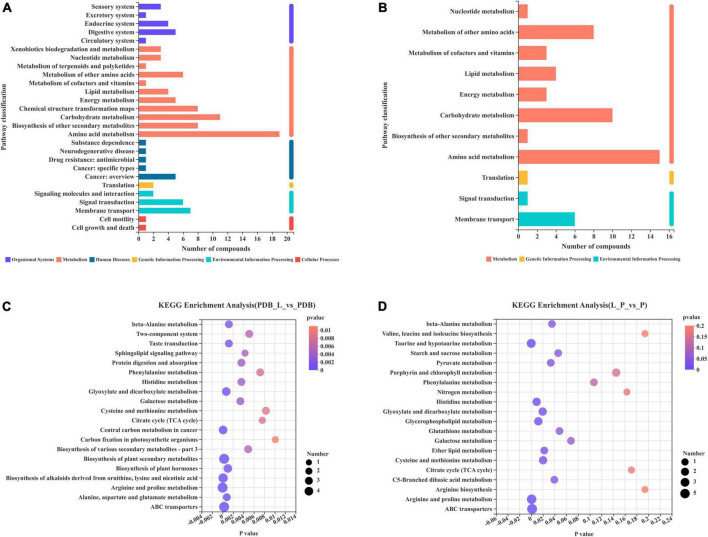
Differential metabolites involved in each metabolic pathway and the number of metabolites. The 20 most abundant metabolic pathways enriched by KEGG **(A,C)** PDB_L vs. PDB groups; **(B,D)** L_P vs. P groups.

The functional annotation statistics of the metabolic pathways of differential metabolites in the PDB_L vs. PDB groups (Figure 8A) and the L_P vs. P groups (Figure 8B), the ordinate is the second classification of the Kyoto Encyclopedia of Genes and Genomes (KEGG) metabolic pathway. A total of 6 first-category pathways were annotated in the PDB_L vs. PDB groups, 2 cellular processes pathways, 3 environmental information processing pathways, 1 genetic information processing pathway, 5 human diseases pathways, 11 metabolism pathways, and 5 organismal systems pathways. Most metabolites were annotated to the metabolic pathway with 69 differential metabolites. A total of 3 first-category pathways were annotated in the L_P vs. P groups, 2 environmental information processing pathways, 1 genetic information processing pathway, and 8 metabolism pathways. Most metabolites were annotated to the metabolic pathway with 45 differential metabolites. Both groups had the most metabolites annotated to the Amino acid metabolism pathway, respectively, the PDB_L vs. PDB groups had 15 metabolites involved in the amino acid metabolism pathway, and the L_P vs. P groups had 19 metabolites involved in the amino acid metabolism pathway.

In the enrichment bubble charts of metabolic pathways in PDB_L vs. PDB groups (Figure 8C) and L_P vs. P groups (Figure 8D), the abscissa is the significant *p*-value of enrichment, the smaller the *p*-value, the darker the color of the bubble, and the more significant the enrichment; The ordinate is the KEGG pathway. The size of the bubbles in the figure represents the number of metabolites in the pathway that are enriched in the metabolic set. It can be seen from Figure 8C that the most influential pathways in the PDB_L vs. PDB groups are arginine and proline metabolism, central carbon metabolism in cancer, biosynthesis of alkaloids derived from ornithine, lysine and nicotinic acid, biosynthesis of plant secondary metabolites, and ABC transporters (*p* < 0.0003). It can be seen from Figure 8D that the most influential pathways in the L_P vs. P groups are taurine and hypotaurine metabolism, arginine and proline metabolism, and ABC transporters (*p* < 0.03). Arginine acts as a link between cellular metabolism and signaling in bacteria, and proline stabilizes proteins and membranes and inhibits protein aggregation during renaturation (Cheng et al., 2016; Barrientos-Moreno et al., 2020). Studies have shown that the production of taurine can induce apoptosis and had the effects of lowering blood lipids and anti-atherosclerosis (Buddhala et al., 2012; Zhang et al., 2016), ABC transporters are directly or indirectly involved in many functions, such as DNA replication, protein degradation, membrane fusion, antibiotic efflux, signal transduction, chemotaxis (Mai-Prochnow et al., 2015; Chen Q. et al., 2021). D-tartaric acid existed in the glyoxylate and dicarboxylate metabolism (*p* = 0.0195) pathway. As an organic acid, it can block the transport of fungal cell membranes, hinder the absorption of nutrients and discharge of waste, and disrupt the balance of the internal and external environment of cells. It can also penetrate the cell to form a destructive force (Kang et al., 2022). After entering the cytoplasm, H+ carried by it will be released due to the high pH value. After the co-culture of *W. cibaria* BYL4.2-CFS and *P. chrysogenum*, the content of D-tartaric acid increased significantly, which increased the content of H+ in the cytoplasm and formed a low pH environment. At the same time, the content of choline sulfate, maltotriose, trans-4-hydroxy-l-proline, taurine, and sucrose in the ABC transporters pathway was reduced, which can indirectly lead to DNA replication, protein degradation, membrane fusion, signal transduction are disrupted, microbial realkalization of the cytoplasm is inhibited, consequently, the growth of *P. chrysogenum* is disrupted.

## Conclusion

In conclusion, *W. cibaria* BYL4.2 had certain bacteriostatic activity against pathogenic bacteria and demonstrated good probiotic properties. *W. cibaria* BYL4.2 has antifungal activity against *P. chrysogenum*, characterization of antifungal activity of CFS showed that the supernatant of the strain was pH-dependent, partially heat-sensitive, and unaffected by protein treatment. LC-MS-based non-targeted metabolomics study to evaluate the impact of metabolite content and pathways among *W. cibaria* BYL4.2, *P. chrysogenum*, and to deeply study their specific reaction processes and inhibitory mechanisms. According to the above results, it is inferred that the main antifungal substance is D-tartaric acid. Overall, the results of the study are an in-depth understanding of the role of *W. cibaria* BYL4.2 in inhibiting fungi and provide new ideas for the mechanism of inhibiting fungal metabolism, which is helpful to supplement and enrich the research on the metabolic mechanism of LAB inhibiting fungi.

## Data availability statement

The datasets presented in this study can be found in online repositories. The names of the repository/repositories and accession number(s) can be found below: NCBI, accession number is OP107888.

## Author contributions

DY and XW designed the experiments, performed most of the experiments, and wrote the manuscript. LM and MW performed the statistical analysis. LX and QY provided valuable advice on experiments design and data analysis tools. DY, LZ, and XZ were the coordinators and guarantors, and oversaw all aspects of this study. All authors read and approved the final manuscript.

## Footnotes

1
https://cloud.majorbio.com/
