# Supplementary material for: Impact of Weissella cibaria BYL4.2 and its supernatants on Penicillium chrysogenum metabolism
Source: Front Microbiol. 2022 Oct 5;13:983613. doi: 10.3389/fmicb.2022.983613 (PMC9581191; doi:10.3389/fmicb.2022.983613)
Supplement: Supplementary file 1 [file Table_1.DOCX]

Supplementary Material

# Supplementary Figures and Tables

**Table S1**

| Sample | Mode | Component | Type | R^2^X | R^2^X(cum) | R^2^Y | R^2^Y(cum) | Q^2^ | Q^2^(cum) |
| --- | --- | --- | --- | --- | --- | --- | --- | --- | --- |
| PDB_L vs PDB | ESI+ | pc1 | PLS-DA | 0.881 | 0.881 | 1 | 1 | 0.999 | 0.999 |
|  | ESI+ | pc2 | PLS-DA | 0.0149 | 0.896 | 0.000243 | 1 | 0.313 | 1 |
|  | ESI- | pc1 | PLS-DA | 0.787 | 0.787 | 0.999 | 0.999 | 0.997 | 0.997 |
|  | ESI- | pc2 | PLS-DA | 0.0324 | 0.819 | 0.000923 | 1 | 0.354 | 0.998 |
|  | ESI+ | pc1 | PCA | 0.698 | 0.698 |  |  |  |  |
|  | ESI+ | pc2 | PCA | 0.0536 | 0.751 |  |  |  |  |
|  | ESI- | pc1 | PCA | 0.628 | 0.628 |  |  |  |  |
|  | ESI- | pc2 | PCA | 0.0628 | 0.691 |  |  |  |  |
| L_P vs P | ESI+ | pc1 | PLS-DA | 0.773 | 0.773 | 1 | 1 | 0.998 | 0.998 |
|  | ESI+ | pc2 | PLS-DA | 0.029 | 0.802 | 0.000058 | 1 | 0.359 | 0.999 |
|  | ESI- | pc1 | PLS-DA | 0.849 | 0.849 | 1 | 1 | 0.999 | 0.999 |
|  | ESI- | pc2 | PLS-DA | 0.0247 | 0.873 | 9.62E-05 | 1 | 0.381 | 1 |
|  | ESI+ | pc1 | PCA | 0.675 | 0.675 |  |  |  |  |
|  | ESI+ | pc2 | PCA | 0.0493 | 0.724 |  |  |  |  |
|  | ESI- | pc1 | PCA | 0.777 | 0.777 |  |  |  |  |
|  | ESI- | pc2 | PCA | 0.0451 | 0.822 |  |  |  |  |

Significance values for the PCA model and PLS-DA models (PDB_L and PDB groups , L_P and P groups).Abbreviations: PDB, PDB; *W. cibaria* BYL4.2-CFS + PDB, PDB_L; *P. chrysogenum*, P; *W. cibaria* BYL4.2-CFS + *P. chrysogenum*, L_P.

**Table S2**

Unique metabolites in PDB_L and L_P, presumed to be partial metabolites of *W. cibaria* -CFS.

**Table S3**

Differentially metabolites identified by LC-MS of PDB_L vs PDB groups.

**Table S4**

Differentially metabolites identified by LC-MS of L_P vs P groups.
